# Effective Removal of Cu^2+^ Ions from Aqueous Media Using Poly(acrylamide-co-itaconic acid) Hydrogels in a Semi-Continuous Process

**DOI:** 10.3390/gels9090702

**Published:** 2023-08-30

**Authors:** Jorge Alberto Cortes Ortega, Jacobo Hernández-Montelongo, Rosaura Hernández-Montelongo, Abraham Gabriel Alvarado Mendoza

**Affiliations:** 1 Department of Chemistry, University Center of Exact Sciences and Engineering, University of Guadalajara, Blvd. Marcelino García Barragán #1421, Guadalajara 44430, Mexico; jorge.cortega@academicos.udg.mx; 2 Department of Physical and Mathematical Sciences, Faculty of Engineering, Catholic University of Temuco, Av. Rudecindo Ortega #2959, Temuco 4813302, Chile; jacobo.hernandez@uct.cl; 3 Department of Translational Bioengineering, University Center of Exact Sciences and Engineering, University of Guadalajara, Blvd. Marcelino García Barragán #1421, Guadalajara 44430, Mexico; rosaura.hernandez@academicos.udg.mx

**Keywords:** semi-continuous process, adsorption by hydrogels, removal Cu^2+^ from wastewater, poly(acrylamide-co-itaconic acid)

## Abstract

Adsorption is one of the most crucial processes in water treatment today. It offers a low-cost solution that does not require specialized equipment or state-of-the-art technology while efficiently removing dissolved contaminants, including heavy metals. This process allows for the utilization of natural or artificial adsorbents or a combination of both. In this context, polymeric materials play a fundamental role, as they enable the development of adsorbent materials using biopolymers and synthetic polymers. The latter can be used multiple times and can absorb large amounts of water per gram of polymer. This paper focuses on utilizing adsorption through hydrogels composed of poly(acrylamide-co-itaconic acid) for removing Cu^2+^ ions dissolved in aqueous media in a semi-continuous process. The synthesized hydrogels were first immersed in 0.1 M NaOH aqueous solutions, enabling OH^−^ ions to enter the gel matrix and incorporate into the polymer surface. Consequently, the copper ions were recovered as Cu(OH)_2_ on the surface of the hydrogel rather than within it, allowing the solid precipitates to be easily separated by decantation. Remarkably, the hydrogels demonstrated an impressive 98% removal efficiency of the ions from the solution in unstirred conditions at 30 °C within 48 h. A subsequent study involved a serial process, demonstrating the hydrogels’ reusability for up to eight cycles while maintaining their Cu2+ ion recovery capacity above 80%. Additionally, these hydrogels showcased their capability to remove Cu^2+^ ions even from media with ion concentrations below 100 ppm.

## 1. Introduction

In recent years, the utilization of heavy metals has witnessed a significant increase due to their participation in numerous industrial processes and their incorporation into various products, devices, and equipment developed to enhance people’s quality of life, as seen in electronics, for example. However, the excessive use of heavy metals has led to dangerous concentrations of these elements in the soil, air, and water. This represents a serious health problem not only for human beings but also for plants and animals, as heavy metals are non-biodegradable and accumulate within the bodies of living organisms, causing poisoning, gastrointestinal and pulmonary diseases, cancer, and cell abnormalities. Prolonged and severe exposure to these metals can even lead to death [1].

Although some heavy metals serve important biological functions in plants and animals, an increase in their concentration, along with their coordination and oxidation-reduction chemical behavior, leads to serious issues. For instance, in humans, copper is an essential element for various organism functions, including physiological processes, immune system functions, fetal and infant development and growth, brain function, bone strengthening, glucose metabolism, iron and cholesterol regulation, among others [2]. However, the excessive presence of this metal in the human body can cause severe health damage. The initial symptoms that usually subside upon reducing exposure to this metal include nausea, abdominal pain, vomiting, and diarrhea [3]. Prolonged exposure to high copper concentrations has been linked to various conditions such as cancer, dementia, Parkinson’s disease, Alzheimer’s, childhood cirrhosis, Wilson’s disease, kidney disease, cell toxicity, among others [4,5]. The daily intake of copper is determined by one’s diet, supplements, and primarily the water ingested. The World Health Organization reported that nearly 104 countries have established an average value of 1.5 mg/L of copper in drinking water. However, copper is widely used in the manufacturing of structural materials, pipes, electronics, heat transfer equipment, the automotive industry, and numerous industrial processes and products, including mining, electroplating, paints, tanneries, and even in the production of fertilizers and pesticides [6]. Consequently, the concentration of this metal has increased in both surface water and groundwater due to these activities.

Due to these concerns, considerable efforts have been made to develop materials and methods capable of removing heavy metals and other pollutants from industrial effluents and water sources. Various methods have been explored, including electrochemical treatments, physicochemical processes such as chemical precipitation and adsorption, as well as more recent advancements such as filtration processes through membranes and photocatalysis [7]. Among these techniques, chemical precipitation stands out as a widely used and economically viable method for heavy metal removal at the industrial level [8,9]. It basically consists of converting a soluble ion into an insoluble compound through a chemical reaction, for example, the formation of metal sulfides, carbonates, and hydroxides. Finally, the insoluble compound is removed from the medium by sedimentation or filtration. In this method, the pH of the medium plays an important role in the recovery of metal ions, and generally, values of pH = 11 are required to increase the amount of metal ions removed [10]. Unfortunately, the chemical substances used and their high concentration necessary in the precipitation process, as well as the sludge obtained that requires certain treatments, can represent a new contamination problem. In that sense, the effectiveness of the chemical precipitation method depends on the type of dissolved ion, its concentration, the precipitating agent, the medium, and the presence of other compounds that can inhibit the reaction [11]. Consequently, this method may not be very effective for low ion concentrations or cases where sedimentation is challenging.

On the other hand, the adsorption process is renowned for its successful application in cases where the concentration of metal ions is low, leading to high-quality treated effluents [12]. This method is not only cost-effective but also highly efficient and easily reproducible and operable. Adsorption relies on mass transfer between the liquid phase and the solid phase (adsorbent), where the negatively charged functional groups on the adsorbent’s surface attract positively charged metal ions [13]. A wide variety of adsorbent materials have been developed, including activated carbon, graphene, carbon nanotubes, zeolites, mesoporous silica, clay, biomass, and hydrogels [14,15]. Among these, hydrogels have garnered the most interest.

Hydrogels are three-dimensional networks composed of synthetic or natural polymer chains containing hydrophilic groups. This characteristic enables them to absorb significant amounts of water while maintaining their shape through physical or chemical crosslinking between the chains. These unique properties have sparked significant interest across various fields of application over the last sixty years. For instance, hydrogels have found applications in the biomedical field [16], tissue engineering [17,18,19,20,21], drug transport [22], agriculture [23], and the removal of heavy metals. In the context of heavy metal removal, hydrogels have proven successful in eliminating metal ions such as copper [24,25], nickel [26,27], lead [28], arsenic [29], cadmium [30], chromium [31], among others.

Considering the significance of chemical precipitation and adsorption methods using hydrogels, this work presents a study focused on the effective removal of Cu^2+^ ions from aqueous media using poly(acrylamide-co-itaconic acid) hydrogels in a semi-continuous process. The objective is to determine the optimal ratio of Cu^2+^ ions to hydroxyl groups that enable the removal of the maximum number of ions, resulting in Cu(OH)_2_ precipitation in the aqueous medium. In this approach, hydrogels serve as transport media for the OH^−^ ions, preventing metal ions from permeating and becoming trapped within the hydrogel matrix. Consequently, the Cu(OH)_2_ formed is located on the hydrogel’s surface, facilitating the removal and recovery of Cu^2+^ ions from the aqueous solution. The application of this method for removing Cu^2+^ or other metallic ions, performed on the surface of the hydrogel, has not been previously documented in the literature. Finally, the hydrogels were employed in a semi-continuous process, wherein the concentration of metal ions gradually decreased. This approach allowed us to assess the number of times the hydrogels could be reused and determine if their removal capacity remained consistent throughout.

## 2. Results

### 2.1. Conversion

**Figure 1:**
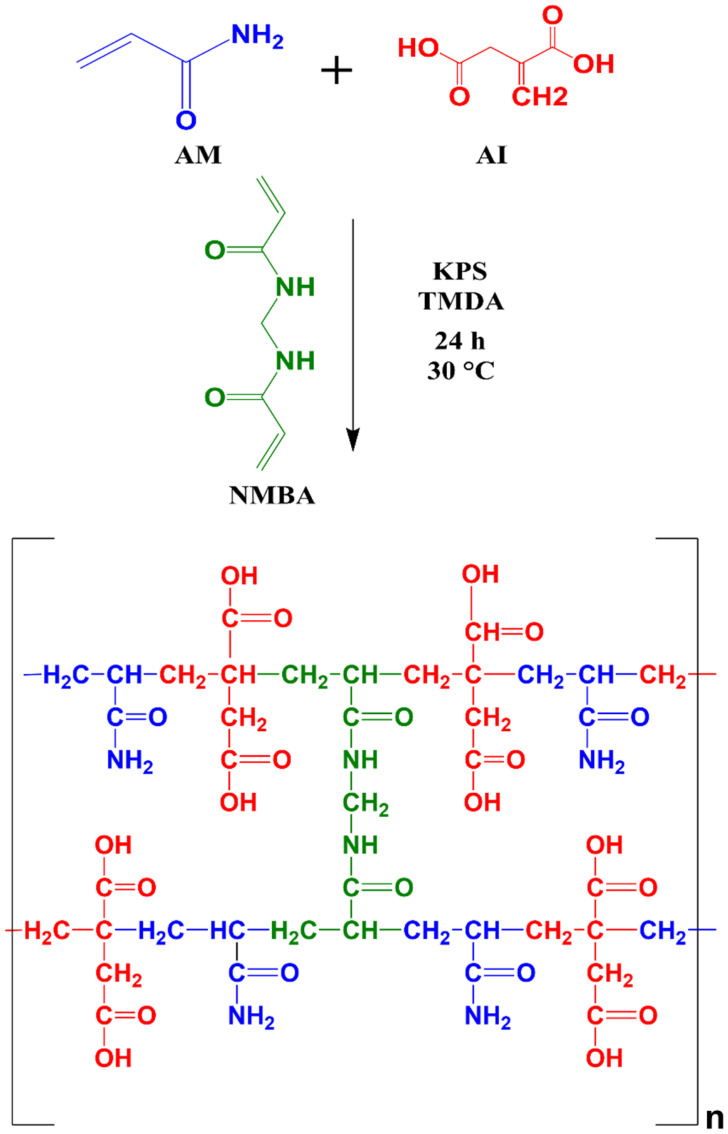
Graphic representation of the network obtained in the copolymerization of AM and AI by means of NMBA. The mass ratio between monomers was not considered.

The hydrogels obtained after the reaction time were smooth and completely solid, without any residues of the aqueous solution from the reaction mixture. The conversion achieved in the synthesized hydrogels was 97 ± 2%, indicating that the reaction was nearly complete. Figure 1 depicts a pictogram illustrating the possible reaction scheme in the synthesis of the copolymer.

### 2.2. Metal Ion Recovery

Table 1 presents the values of the mass of the 0.1 M NaOH solution (mass_NaOH_) and the mass of the mixed CuCl_2_∗2H_2_O solution (mass_CuCl2_), along with the percentage of copper ions removed (R_Cu_^2+^_)_ and the molar ratio between the OH^−^ and Cu^2+^ groups (mol_OH_^−^/mol_Cu_^2+^).

It was observed that when the mol_OH_^−^/mol_Cu_^2+^ ratio reached the value of 2.74, the percentage of copper ions removed was 97.9%. Beyond this ratio, no significant increase in metal removal was observed, even with an increase in the amount of NaOH solution added. In Experiments 7 and 8, the removal percentage decreased. Hence, it was determined that the optimal value for the mol_OH_^−^/mol_Cu_^2+^ ratio was 2.74. Regarding the adsorption process through the use of hydrogels, maintaining a ratio of 2.74 between the moles of OH^−^ present within the hydrogel matrix and the concentration of Cu^2+^ ions in the reaction medium resulted in a Cu^2+^ removal efficiency (Re_Cu_^2+^) of 98.52%. Removal efficiency through the chemical precipitation method was equaled, enabling us to pinpoint the optimal ratio of mol_OH_^−^/mol_Cu_^2+^ for attaining the utmost elimination of Cu^2+^ ions. Furthermore, this process can be repeated multiple times using the same hydrogel, as the precipitate formed on the surface of the hydrogel can be easily removed by gently shaking the medium and separating it by decantation. The hydrogel can be regenerated by reintroducing it into a NaOH solution, making it suitable for reuse and thereby reducing operating costs.

To evaluate the effect of the concentration of the NaOH solutions in which the xerogels are immersed and swollen, they were immersed in solutions with concentrations of 0.1, 0.2, 0.3, and 0.4 M. It was found that the swelling capacity (W (%)) of the hydrogels decreased as the concentration of the NaOH solution increased, ranging from 64% to 50%. This decrease is attributed to the higher presence of Na^+^ and OH^−^ ions in the medium. Consequently, the interaction between water molecules and the hydrophilic chains of the polymer diminishes, resulting in a reduction in its swelling.

Subsequently, when the hydrogels swollen with the NaOH solution were immersed in the CuCl_2_∗2H_2_O solution with 1000 ppm Cu^2+^ at a ratio of Cu^2+^ ion solution mass to xerogel mass of 200/1, it was observed that the mass of copper removal per gram of xerogel (mg_Cu_^2+^/g_xerogel_) slightly increased with the increasing concentration in NaOH solutions, from 0.1 M (183 mg_Cu_^2+^/g_xerogel_) to 0.4 M (211 mg_Cu_^2+^_/_g_xeroge_l). Additionally, the R_Cu_^2+^ (%) increased from 98% to 99.7%. Table 2 summarizes the values of the swelling capacity, mg_Cu_^2+^/g_xerogel_ removed, and the R_Cu_^2+^ as a function of the concentration of the NaOH solution.

The R_Cu_^2+^ values were very similar in all cases. This is because the metal removal occurs through the reaction of the OH^−^ ions present inside the gel, which migrate toward the gel’s surface and react with the metal ions. The efficiency of the removal was not dependent on the concentration of the NaOH solutions but on the amount of OH^−^ ions within the hydrogels. Therefore, as the absorption capacity of the hydrogels decreased due to the increase in NaOH concentration, the OH^−^ ions inside the hydrogel also decreased, resulting in similar efficiency in copper recovery in all cases. Thus, a 0.1 M concentration of NaOH in the solution was the most suitable for the recovery of copper ions present in the solution, similar to the precipitation method. This proves that hydrogel functions akin to a “sponge,” proficient in capturing, transporting, and releasing OH^−^ ions. In that sense, the AI played an important role due to its ability to confer carboxylic groups to the hydrogel. Such groups facilitate pronounced swelling in basic environments, thereby enhancing the influx of OH^−^ ions when diluted solutions of NaOH were employed [32].

**Figure 2:**
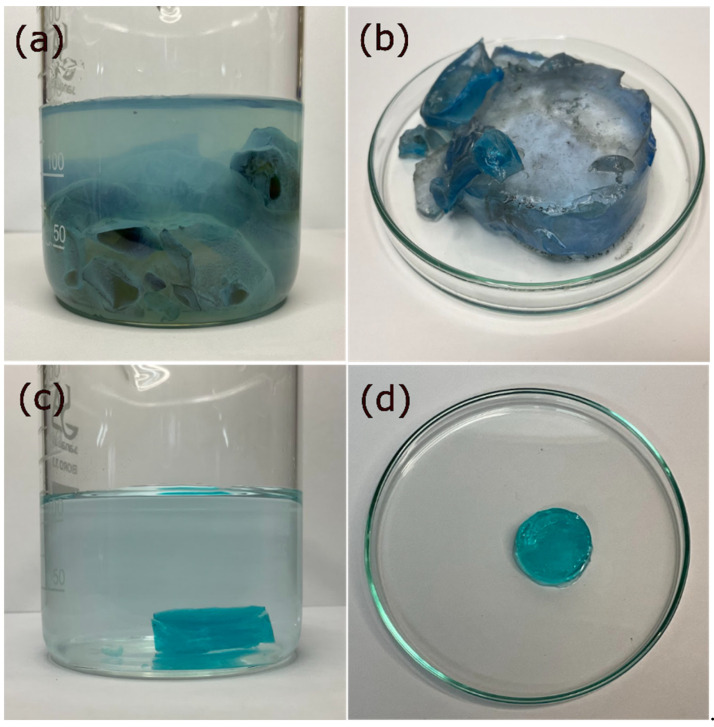
The xerogels immersed in NaOH solutions removed Cu^2+^ in the form of Cu(OH)_2_, which forms on its surface (**a**). Only a small part of these ions managed to enter the matrix; thus, the hydrogel remained swollen (**b**). In contrast, in the case of water-swollen xerogels, (**c**) Cu^2+^ ions entered the matrix, causing the hydrogel to collapse (**d**).

To verify this, the process was carried out by immersing the xerogels in bidistilled water under the same conditions mentioned above and in the same proportion. Subsequently, they were placed in 1000 ppm Cu^2+^ solutions at a ratio of Cu^2+^ solution mass to xerogel mass equal to 200/1. In this case, the ions were not recovered on the surface but within the gel matrix. The cations penetrated the gel matrix, causing it to saturate, and as a result, the network closed, leading to the collapse of the hydrogel. Additionally, as seen in the residual water, Cu^2+^ ions were still present (Figure 2).

Moreover, when xerogels were directly introduced into a 1000 ppm Cu^2+^ solution, without a previous immersion in 0.1 M NaOH solution, at a mass ratio of 200/1 maintained at 30 °C without agitation and left for a duration of 48 h, the R_Cu_^2+^ value equated to 14.67% or 29.29 mg Cu^2+^/g of xerogel. This is only 16% of the obtained R_Cu_^2+^ compared to the previous immersion in NaOH: 98.52% or 183 mg Cu^2+^/g of xerogel, which confirms the key role of OH^−^ ions in the process.

**Figure 3:**
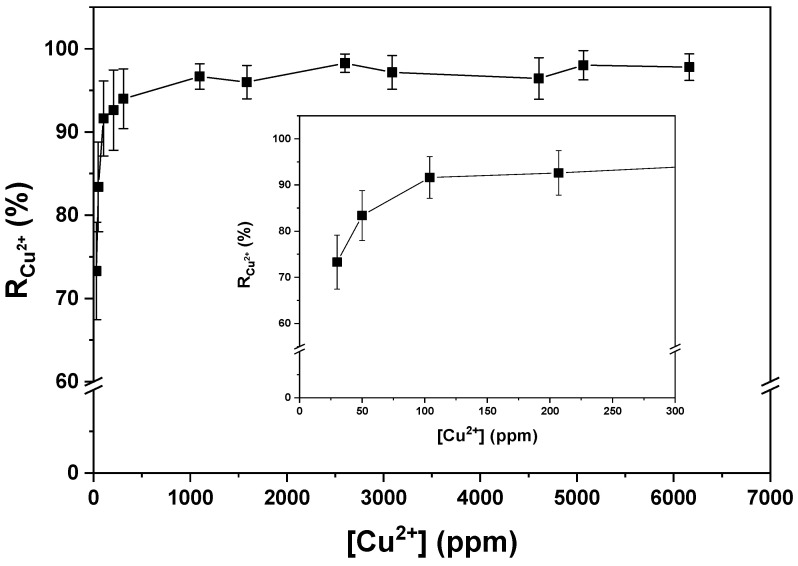
The amount of Cu^2+^ removed through the use of hydrogels while maintaining a ratio of mol_NaOH_/mol_Cu_^2+^ equal to 2.74, as the total volume of the Cu^2+^ ion solution is increased.

**Figure 4:**
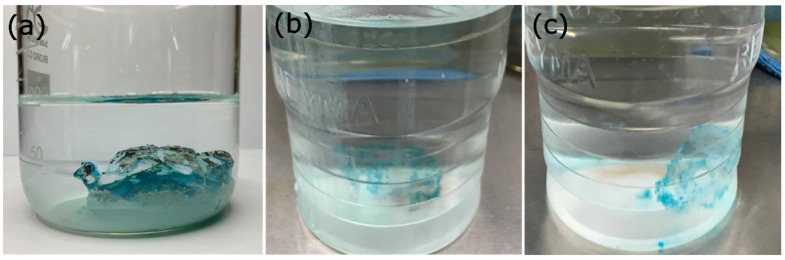
Recovery of copper ions in aqueous solutions of cupric chloride at different concentrations: 30 (**a**), 20 (**b**), and 10 ppm (**c**), while maintaining a ratio of mol_NaOH_/mol_Cu_^2+^ at the value of 2.74.

In another experiment, it was demonstrated that if the total volume of the metal ion solution is increased while keeping the mol_OH_^−^/mol_Cu_^2+^ ratio constant at the value of 2.74, the R_Cu_^2+^ value remains around 97% with no significant changes until a concentration of 1000 ppm (Figure 3). Below that concentration, the R_Cu_^2+^ value decreased to about 75% for concentrations of 30 ppm (inset Figure 3). Another study was conducted to determine the detection limit of Cu^2+^ ions by the hydrogel, and it was found that this process can cause the precipitation of metal ions in solutions containing up to 10 ppm Cu^2+^ (Figure 4c). The value of R_Cu_^2+^ was not displayed for concentrations lower than 30 ppm due to reading and precision limitations in UV-vis analysis. On the other hand, it can be observed that the standard deviation was much higher for concentrations lower than 100 ppm due to reading limitations and the precision obtained with the technique used in the analysis. Figure 4 shows photographs of the experiment, where the formation of Cu(OH)_2_ on the surface of the hydrogel can be observed in solutions with concentrations of 30, 20, and 10 ppm.

**Figure 5:**
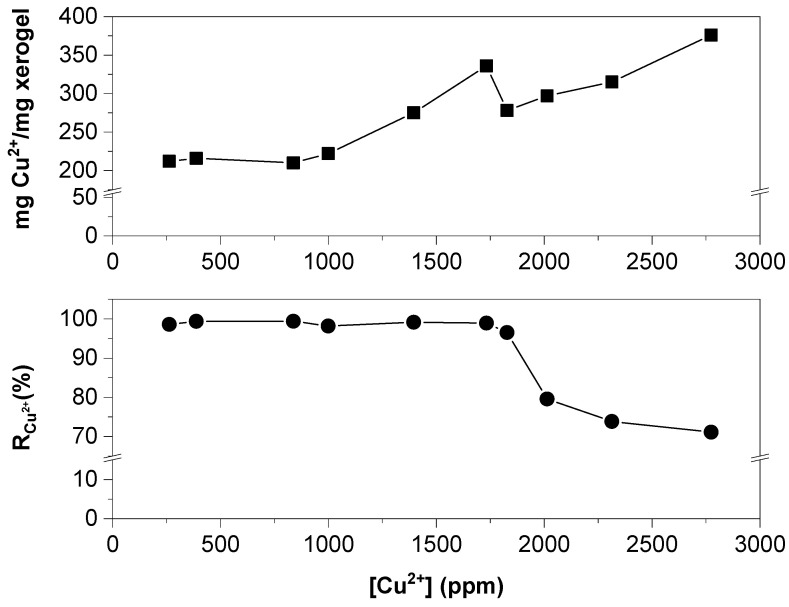
Effect of the initial concentration of Cu^2+^ ions on the R_Cu_^2+^ and mg of Cu^2+^/g of xerogel.

The effect of the initial concentration of dissolved Cu^2+^ ions on the R_Cu_^2+^ and the value of mg Cu^+2^/g of xerogel are shown in Figure 5. It was observed that as the concentration of initial cations in the medium increased, the value of R_Cu_^2+^ remained, on average, at 98.61% for concentrations ranging from 200 to 1750 ppm. However, for concentrations greater than 1750 ppm, the R_Cu_^2+^ value gradually decreased, falling below 80% for initial concentrations higher than 2000 ppm. This decline is attributed to the insufficient amount of OH^−^ ions present inside the hydrogel to effectively react with the high concentration of Cu^2+^ ions. In the range of concentrations where the R_Cu_^2+^ was greater than 95%, the mol_OH_^−^/mol_Cu_^2+^ ratio was found to be higher than 2.74. Conversely, for values where the R_Cu_^2+^ decreases below 80%, the mol_OH_^−^/mol_Cu_^2+^ ratio was less than 2.74. Therefore, the two most important parameters for effective copper ion removal were the initial cation concentration and the amount of OH^−^ ions present within the hydrogel.

Regarding the maximum mg Cu^+2^/g of xerogel ratio obtained within acceptable R_Cu_^2+^ values, it was 336 mg Cu^+2^/g of xerogel. Although in higher initial concentrations, the maximum ratio of mg Cu^+2^/g of xerogel was also 336 mg of mg Cu^+2^/g of xerogel, the R_Cu_^2+^ was no longer acceptable because it reached only 71%.

**Figure 6:**
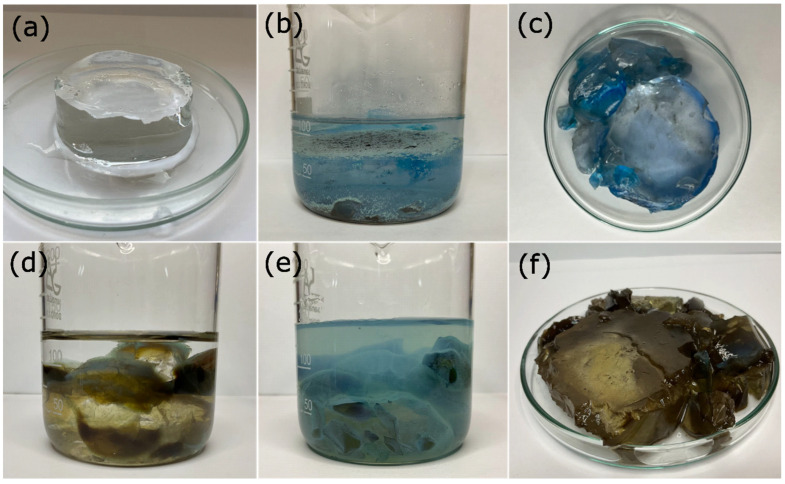
Semi-continuous metal ion removal process: obtained xerogel swollen after immersion in a 0.1 M NaOH solution (**a**), hydrogel immersed in the Cu^2+^ ion solution (**b**), hydrogel after the first stage (**c**), hydrogel regeneration in the NaOH solution (**d**), hydrogel immersed in ion solution Cu^2+^ (**e**), and hydrogel after being used eight times (**f**).

Finally, in the semi-continuous study, it was found that the same hydrogel sample can be used up to eight times (Figure 6). The process was categorized as a semi-continuous process because the hydrogel required a retention time at each stage to facilitate the elimination of Cu^2+^ ions (which constitutes a batch process). Following this, the hydrogel was regenerated and proceeded to the subsequent stage, wherein the concentration of Cu^2+^ ions was lower compared to the previous stage (representing a continuous process).

**Figure 7:**
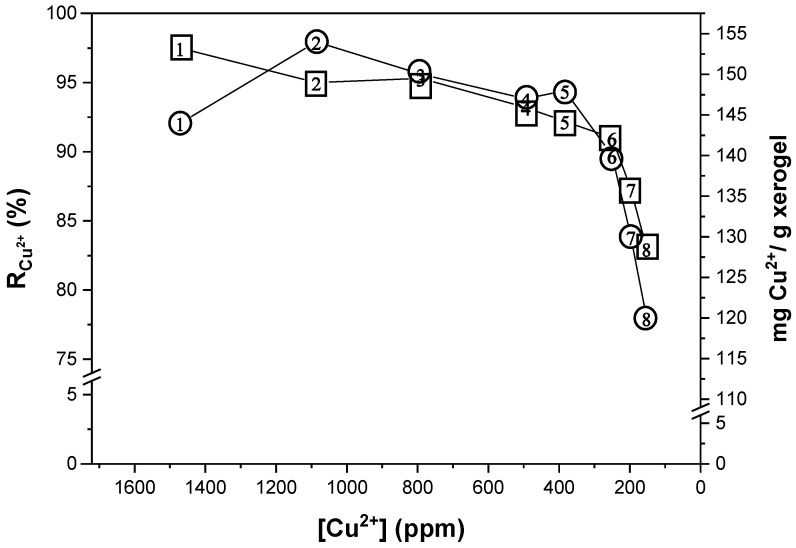
Values of R_Cu_^2+^ (□) and mg_Cu_^2+^/g_xerogel_ (○) as a function of the initial concentration of Cu^2+^ ions in each stage.

On average, the R_Cu_^2+^ value was 93.40% in the first six stages, subsequently decreasing to 87.30% and finally to 83% in the last stage (Figure 7). This decrease in removal capacity is attributed to the fact that in each stage where the hydrogel was used, a small fraction of the metal ions remain trapped in its matrix (Figure 6c,f), which accumulated with each reuse. As a result, the amount of OH^−^ ions inside the hydrogel decreased, leading to a reduction in its ability to remove the cations. Furthermore, the lowest R_Cu_^2+^ value was found in solutions with the lowest amount of initial Cu^2+^ ions. As previously demonstrated, the two main factors for maintaining high R_Cu_^2+^ values were the concentration of OH^−^ ions in the hydrogel and the initial concentration of Cu^2+^ ions in the solution.

The achieved percentage removal of copper ions using this method was 98.52% at a temperature of 30 °C with a residence time of 48 h, no agitation, and with a mass ratio of Cu^2+^ solution to xerogel of 200/1. This corresponds to the maximum removal capacity (Qmax) of 183 mg Cu2+/g xerogel, recovered on the surface of the hydrogel in the form of Cu(OH)2, which is easily removed by slightly shaking the container or simply rinsing with the minimum amount of distilled water to the hydrogel. This avoids the use of acidic solutions for the recovery of the metal ions, a practice employed in processes in which the metal ions remain within the gel matrix. Table 3 provides a compilation of various adsorption studies along with their respective Qmax values. While this study does not boast the highest Qmax value, it does mark the pioneering utilization of the hydrogel surface for recovery. Furthermore, it shows a method for the easy recovery of metal ions in a process similar to industrial water treatment, where the concentration of the ions decreases in the process, deviating from the conventional practice of recovering throughout the entire matrix.

## 3. Conclusions

The present work demonstrated that hydrogels composed of poly(acrylamide-co-itaconic acid) were capable, efficient, and economical for the removal of Cu^2+^ ions from aqueous solutions. Once the xerogels were swollen in 0.1 M NaOH aqueous solutions, they acted as carriers for OH^−^ ions, which reacted with dispersed Cu^2+^ ions in the solution to form Cu(OH)_2_. This Cu(OH)_2_ adhered to the hydrogel surface, preventing its dispersion in the solution. The supernatant-diluted solution was removed by decantation, and the solid formed was recovered by rinsing the hydrogel. The hydrogel was regenerated by submerging it again in 0.1 M NaOH solutions and was used up to eight times while maintaining its removal capacity above 80%. It was demonstrated that when the mol_OH_^−^/mol_Cu_^2+^ ratio was equal to 2.74, the R_Cu_^2+^ was 98%. As long as this ratio was maintained, the total volume of the solution in which the hydrogel was immersed did not affect its cation removal capacity. Based on the experiments carried out, the factors that greatly influenced the removal capacity of metal ions were the amount of OH^−^ ions inside the hydrogels and the initial concentration of Cu^2+^ ions in the medium. Additionally, the maximum mg_Cu_^2+^/g_xerogel_ ratio obtained was 336 when the initial concentration of Cu^2+^ was 1750 ppm, with an average value of W = 64.24%. Finally, it was demonstrated that this process can be used in very diluted solutions as 30, 20, and 10 ppm of Cu^2+^.

## 4. Materials and Methods

### 4.1. Materials

The monomers acrylamide (AM), itaconic acid (AI), and the CuCl_2_∗2H_2_O salt, all with a purity of 99%, were obtained from Aldrich (St. Louis, MO, USA). The initiator used in the polymerizations was potassium persulfate (K_2_S_2_O_8_) (KPS), also with a purity of 99%, sourced from Aldrich (St. Louis, MO, USA), along with the crosslinking agent N,N′-methylenebisacrylamide (NMBA). To carry out the polymerization reactions at 30 °C, N,N,N′N′ groups (mol-tetramethyl-ethylenediamine (TMDA) from Tokyo Kasei (Shanghai, China) served as an accelerator. Finally, sodium hydroxide (NaOH) with 99% purity from Aldrich (St. Louis, MO, USA) and bidistilled water from Productos Selectropura (Guadalajara, Mexico) (pH = 6.36) were used as the reaction medium. All reagents were used as received.

### 4.2. Hydrogel Synthesis Reactions

The synthesis reactions were conducted in glass vials under temperature control using a LAUDA Eco Silver (LAUDA DR. R. WOBSER GMBH & CO. KG, Germany) brand overboard thermostat set at 30 °C, with a reaction time of 24 h. The composition of the reaction mixture in all cases was 90% water by mass and 10% monomers by mass, with a mass ratio of 80/20 AM/AI (0.1125/0.0154 molar ratio). For every total mass of monomers, 1% KPS, 2% TMDA, and 1% NMBA were added by mass (percentage molar ratio corresponds to 0.289 KPS, 0.5074 NMBA, and 1.3448 TMDA with respect to the total amount of monomers). Afterward, the hydrogels were removed from the vials and cut into 0.5 cm-thick discs, identified with three sections: upper, middle, and lower. Subsequently, the hydrogels were immersed in bidistilled water to wash and eliminate all traces of the reaction. The water was replaced every 6 h for 3 days and then every 24 h for a further 5 days. Previous research has demonstrated that this process is sufficient for cleaning the materials [42].

### 4.3. Conversion Determination

To measure the conversion, the hydrogels were removed from the vials and cut into 0.5 cm-thick discs. These discs were then placed in Teflon Petri dishes and subjected to a convection oven at 50 °C until a constant mass was achieved, resulting in xerogels (completely dry hydrogel). Subsequently, the xerogels were immersed in bidistilled water to clean the hydrogels following the previously described procedure. Finally, the samples were placed back in the drying oven until they reached a constant mass again. The yield of the reaction was determined by gravimetry using the following equation:

(1) $$X\%=\frac{M_{x,0}-M_{x,t}}{M_{x,0}}\times 100$$

where *M*_*x*,0_ is the mass of the xerogel before being washed, and *M_x,t_* is the mass of the xerogel after undergoing the washing process.

### 4.4. Batch Study of Removal of Cu^2+^ Ions in Aqueous Solution

First, in order to determine the optimum ratio of moles of OH^−^ used to moles of Cu^2+^ present (mol_OH_^−^/mol_Cu_^2+^) for enhanced metal ion precipitation, solutions of CuCl_2_∗2H_2_O with 1000 ppm of Cu^2+^ were prepared and mixed with aqueous solutions of 0.1 M NaOH in varying proportions. This allowed us to identify the mol_OH_^−^/mol_Cu_^2+^ ratio ranging from 0.71 to 56.36. The residence time for the experiments was 48 h, conducted at a constant temperature of 30 °C. These experiments were performed without gels.

Once the most suitable ratio was determined, the obtained xerogels in Section 4.2 were weighed using an OHAUS (OHAUS CORPORATION, Parsippany, NJ. USA) brand balance with a precision of 0.0001 g. Subsequently, they were immersed in 0.1 M NaOH aqueous solutions for 24 h at 30 °C without stirring, with a mass ratio of NaOH solution/xerogel (NaOH/xerogel) set at 125/1. The amount of NaOH solution absorbed by the hydrogels was calculated using the following equation:

(2) $$W=\frac{m_{t}-m_{0}}{m_{0}}\times 100$$

where *m_t_* is the mass of the hydrogel swollen at time *t*, and *m*_0_ is the mass of the xerogel.

**Figure 8:**
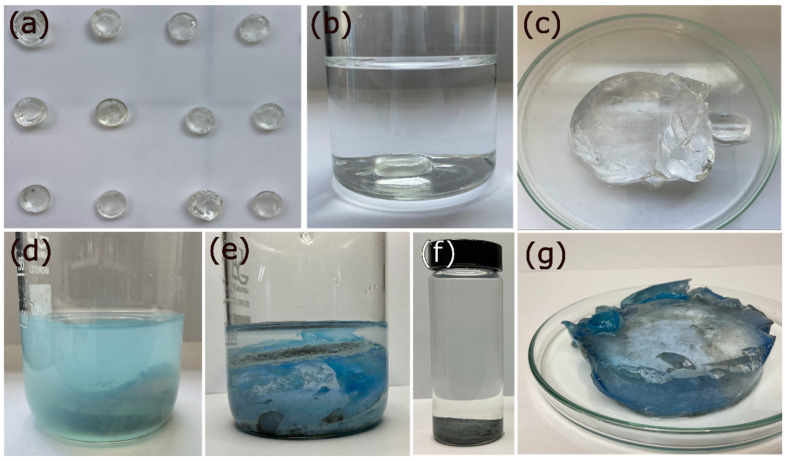
Obtained xerogels (**a**). A xerogel is immersed in a 0.1 M NaOH solution with a mass ratio of 125/1 for 48 h (**b**), resulting in a swollen hydrogel (**c**). The hydrogel is then immersed in a copper ion solution of 1000 ppm for 48 h (**d**), facilitating the migration of copper ions from the solution to the hydrogel (**e**). The generated Cu(OH)_2_ is separated by decantation (**f**), and the hydrogel is subsequently regenerated for reuse (**g**).

After 48 h, the hydrogels were removed from the NaOH solution and placed in CuCl_2_∗2H_2_O solutions containing 1000 ppm Cu^2+^, maintaining the optimal mol_OH_^−^/mol_Cu_^2+^ ratio identified in the chemical precipitation process. The subsequent study focused on evaluating the effect of the concentration of NaOH solutions used to swell the hydrogels, the total volume of copper solution while maintaining a constant mol_OH_^−^/mol_Cu_^2+^ ratio, and the initial concentration of copper ions on the hydrogels’ ability to remove metal ions. The overall process is illustrated in Figure 8.

### 4.5. Semi-Continuous Study of Removal of Cu^2+^ Ions in Aqueous Solution

To determine the reusability of hydrogels for metal ion recovery, the same hydrogel sample was immersed in different CuCl_2_∗2H_2_O solutions, each with a decreasing concentration of Cu^2+^ ions from one container to another (from one stage to another). The process involved removing Cu(OH)_2_ from the hydrogel in the first container, followed by washing with distilled water. Subsequently, the hydrogel was placed back in a 0.1 M NaOH solution at a 125/1 NaOH to xerogel solution ratio. Afterward, this hydrogel was placed in a new aqueous solution of CuCl_2_∗2H_2_O with a mass ratio of Cu^2+^ solution to xerogel of 200/1. The concentration of the metal solution was progressively reduced to simulate a semi-continuous metal ion removal process using the synthesized hydrogels.

In both the batch study and the semi-continuous study, the amount of Cu^2+^ ions removed was quantified using UV-visible spectroscopy (UV-vis). To achieve this, the supernatant of each solution was decanted, and the first 15 mL were utilized for residual copper measurement. A previously established calibration curve on a UV-visible spectrophotometer, UNICO model UV2150 (United Products & Instruments Inc., Dayton, NJ. USA), at the wavelength of 800 nm, aided in determining the concentration of Cu^2+^ ions. The percentage of Cu^2+^ ions removed (R_Cu_^+2^) from the medium was calculated using the following equation:

(3) $$R_{{Cu}^{+2}}(\%)=\frac{\left[{Cu}_{0}^{2+}\;\right]-[{Cu}_{f}^{2+}]\;}{\left[{Cu}_{0}^{2+}\right]}\times 100$$

where $\left[{Cu}_{0}^{2+}\right]$ represents the initial concentration of Cu^2+^ ions, and $\left[{Cu}_{f}^{2+}\right]$ is the residual concentration. Throughout all experiments, five samples were used, and the average values were reported.

## Figures and Tables

**Table 1:** Removal of copper ions as a function of the mass ratio of NaOH solution/copper solution.

Experiment	1	2	3	4	5	6	7	8	9
mass_CuCl2_ (g)	18	16	14	12	10	8	6	4	2
mass_NaOH_ (g)	2	4	6	8	10	12	14	16	18
mol_OH_^−^/mol_Cu_^2+^	0.71	1.59	2.74	4.27	6.37	9.52	14.78	25.37	56.36
R_Cu_^2+^(%)	30	41	98	99	98	99	95	93	98

**Table 2:** Removal of copper ions as a function of the concentration of NaOH solutions, with a lye/xerogel ratio of 125/1 and a copper/xerogel solution of 200/1.

[NaOH] M	W (%)	mg_Cu_^2+^/g_xerogel_	R_Cu_^2+^ (%)
0.1	64	183	98.52
0.2	55	194	98.44
0.3	53	205	99.10
0.4	50	211	99.68

**Table 3:** Adsorption capacities of different adsorbent materials for Cu^2+^ from other studies.

Adsorbents Materials	Q_max_ (mg/g)	Reference
Graphene oxide-polyethylene glycol and polyvinyl alcohol (GO-PEG-PVA) triple network hydrogel	917	[33]
Hybrid hydrogel of acrylic acid monomer/wheat bran/montmorillonite	17.64	[34]
Hydrogels comprised of polysaccharide salecan injerted with poly(3-sulfopropyl methacrylate potassium salt).	107.2	[35]
Aerogels comprised of carboxylated cellulose and MnFe_2_O_4_.	73.70	[36]
Hydrogels comprised of Loess of clay/Itaconic acid/2-Hydroxyethyl methacrylate/N-vinyl-2-pyrrolidone	594.43	[37]
Poly(acrylic acid-co-itaconic acid)/NaOH hydrogel	85	[38]
Polyvinyl alcohol/alginate/iron oxide nanoparticles (PAI) hydrogels	60	[39]
Carboxymethylcellulose sodium/polyvinyl alcohol (PVA)/Cellulose nanocrystals hydrogels	108.8	[40]
Corn starch/acrylic acid/itaconic acid ion exchange hydrogel	699.31	[41]

## Data Availability

The data presented in this study are available on request from the corresponding author.
